# Breaking up prolonged sitting with moderate-intensity walking improves attention and executive function in Qatari females

**DOI:** 10.1371/journal.pone.0219565

**Published:** 2019-07-12

**Authors:** Bryna C. R. Chrismas, Lee Taylor, Anissa Cherif, Suzan Sayegh, Daniel P. Bailey

**Affiliations:** 1 Qatar University, Sport Science Program, College of Arts and Science, Doha, Qatar; 2 ASPETAR, Qatar Orthopaedic and Sports Medicine Hospital, Athlete Health and Performance Research Centre, Doha, Qatar; 3 Loughborough University, School of Sport, Exercise and Health Sciences, Loughborough, United Kingdom; 4 ASPETAR, Qatar Orthopaedic and Sports Medicine Hospital, Exercise is Medicine, Doha, Qatar; 5 Institute for Sport and Physical Activity Research, School of Sport Science and Physical Activity, University of Bedfordshire, Bedford, United Kingdom; University of New Brunswick, CANADA

## Abstract

**Background:**

Cultural, environmental and logistical factors promote a sedentary lifestyle within Qatar, particularly for females. Sedentary behaviour is acutely associated with poor cognitive function and fatigue, and chronically may be implicated with cognitive decline (i.e. Alzheimer’s disease).

**Purpose:**

To examine the effects of breaking up sitting with short-duration frequent walking bouts on cognitive function and fatigue in Qatari females.

**Method:**

Eleven sedentary (sitting ≥7 h/day) females completed three visits; the first being familiarisation. In a cross-over randomised manner, experimental visits two and three were identical, except participants either remained seated for 5-h (SIT) or interrupted their sitting every 30-min with a 3-min moderate-intensity walk (WALK) on a motorised treadmill. The Computerised Mental Performance Assessment System (COMPASS) assessed cognition at baseline (-15-min), and then at 2.5-h and 5-h into the experimental conditions. Specific COMPASS tasks employed were; serial-3 subtractions (2-min), serial-7 subtractions (2-min), simple reaction time (RT; 50 stimuli), rapid visual information processing [RVIP (5-min)], choice reaction time (CRT; 50 stimuli), and Stroop (60 stimuli); and a visual analogue scale for fatigue (VAS-F) was completed at the same time intervals.

**Results:**

There was a significant condition effect for CRT (f = 26.7, p = 0.007). On average CRT was 101 s (95% CI = -47 to -156 s) quicker in WALK compared to SIT. There was a significant time effect for CRT (f = 15.5, p = 0.01). On average CRT was 134 s slower at 5-h compared to baseline (p = 0.006; 95% CI = -64 to -203 s), and 114 s slower at 5-h compared to 2.5-h (p = 0.01; 95% CI = -44 to -183 s). There was a significant interaction effect for RT in the Stroop incongruent task (f = 10.0, p = 0.03). On average RT was 210 s quicker at 2.5-h in WALK compared to SIT (p = 0.01; 95% CI = -76 to -346 s).

**Conclusion:**

Breaking up prolonged sitting with moderate-intensity walking offers an ecologically valid intervention to enhance some aspects of cognitive function, whilst not affecting fatigue in sedentary Qatari females. Whilst these findings are promising, the long-term effects of breaking up sitting on cognitive function requires testing before population level recommendations can be made.

## Introduction

Within Qatar, >70% of the population is either ‘overweight’ or ‘obese’ and 83% participate in little or no physical activity [1]. Furthermore, 44% of Qatari females achieve less than 5,000 steps per day and are thus highly sedentary [2]. Particular cultural barriers, beliefs, values and practices, as well as the climate (i.e. hot desert climate) and physical activity infrastructure, challenge this population to engage with and obtain sufficient physical activity. Indeed, Islamic traditional clothing (i.e. Abaya, Hijab), adopted widely by Qatari females in public places, has been considered an additional barrier regarding engagement with physical activity [3].

The deleterious effects of sedentary behaviour on psychological health and cognitive function remain much less understood compared to the cardiometabolic consequences [4]. A sedentary lifestyle [5] is associated with impaired cognitive function acutely, whilst chronically, evidence is emerging for its complicity within cognitive decline and dementia risk [6]. Breaking up sitting time has been shown to acutely improve cerebral blood flow [7] and lower daily sitting time is beneficially associated with vitality and mental health [8]. Yet, the first edition of the Qatar National Physical Activity guidelines [9] does not contain any specific recommendations on reducing sitting time.

Limited data (n = 13 studies) has examined the cognitive effects of reducing workplace sitting in adults [4] and further understanding of the effects of breaking up sitting on cognitive function is thus needed as prolonged sitting could be associated with reduced efficiency and productivity [10, 11]. Specifically, sit-to-stand desks and active workstations in the workplace have elicited trivial changes in productivity [12]. Furthermore, whilst prolonged standing at work (2-h) improved creative problem solving, it was associated with discomfort and decreased reaction time [13]. Therefore, breaking up prolonged sitting with frequent simple palatable walking breaks may be a more practically viable intervention to enhance cognitive processes [4] and facilitate improvements in cardiometabolic health [14]. Indeed, improvements in cerebral blood flow appear to occur through short-duration regular walking breaks, as opposed to less frequent longer duration walking breaks [7]. Additionally, short-duration (5-min every hour for 6-h) moderate-intensity walking breaks, but not a single continuous 30-min bout of walking, improved mood and decreased fatigue in sedentary adults [15]. However, no research has yet examined the cognitive or fatigue response to breaking up sitting in a Qatari population.

Given the aforementioned environmental and cultural barriers for Qatari females, breaking up prolonged sitting in the workplace with short and frequent walking bouts (i.e. within the air-conditioned office) may be a simple and ecologically valid intervention, which can be performed whilst wearing Islamic traditional clothing. Therefore, the aim of this study was to examine the effects of breaking up prolonged sitting time with short walking breaks on a range of cognitive tests, and fatigue, in Qatari females. It was hypothesised that breaking up sitting would improve (i) cognitive function and (ii) decrease fatigue.

## Methods

### Experimental design

**Fig 1:**
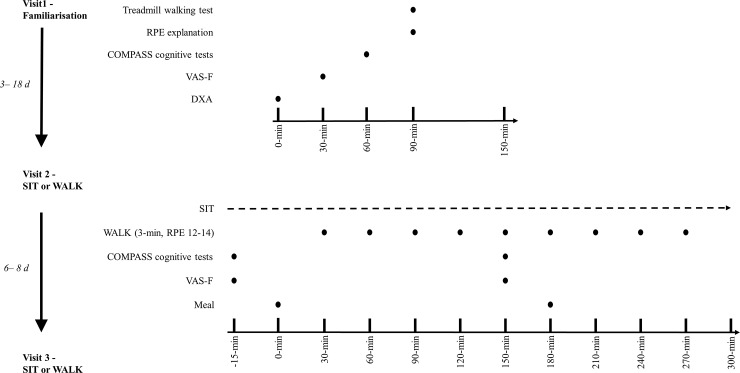
Experimental schematic. SIT = uninterrupted sitting; WALK = breaking up sitting with walking. Both conditions are identical except in SIT, no walking breaks occurred.

Using a randomised (GraphPad online QuickCalcs) crossover design all experimental procedures were conducted within a temperature controlled laboratory (24 ± 0.3°C) at Aspetar Orthopaedic and Sports Medicine Hospital Qatar. The intervention utilised to break up sitting (see Fig 1) has been employed elsewhere [16, 17]. All participants completed three separate visits (familiarisation, experimental visit 1 and 2), with temporality indicated on Fig 1. Participants were recruited from 1^st^ February 2017 to the 20^th^ November 2017. Although unlikely, there may be mood disturbances associated with changes in hormones during menstruation [18], therefore participants completed the experimental visits in their follicular phase. Participants refrained from exercise for 48-h prior to each experimental visit and recorded volume and timings of all food and liquids consumed in the 24-h prior to the first experimental visit in a food diary; this intake was replicated the day before the subsequent visit. Ethical approval was received from the Anti-Doping Laboratory Qatar Institutional Review Board (IRB# F2016000196). Prior to any experimental procedure occurring written informed consent was obtained in the spirit of the Declaration of Helsinki (1975). This study was not registered as a clinical trial. The SPIRIT checklist [19] was followed, excluding the following items which were not applicable; 2–3, 5, 6a and b, 11, 16, 17, 19, 21–33.

### Participants

Eleven sedentary (sitting ≥7 h/day; average 198 MET min/week) Qatari females [median (minimum–maximum) age 27 (21–44) y; height 1.64 (1.57–1.74) m; body mass 57.8 (47.0–87.4) kg; body fat 36 (24–45) %] completed the study. Participants were excluded if they met a single criterion from those outlined in Table 1. Sample size calculations for this study were based on postprandial glucose as the primary outcome (data not reported in this article). In addition to investigating the cardiometabolic effects, we also wanted to explore the potential cognitive function and fatigue responses to the intervention, which are thus reported in this article.

**Table 1:** Participant exclusion criteria.

Currently fasting or expecting to fast/modify dietary habits during the time of participation
Self-reported pregnancy
Diagnosed diabetes
Hypertension
Renal failure
Liver disease
History of severe cardiovascular complications
Body mass index >45 kg/m^2^
Taking glucose or lipid-lowering medication
Smoking
Known physical activity contraindications
Major illness/injury
Other health issues that may limit ability to perform the walking bouts

### Familiarisation (visit 1)

Body composition was analysed using dual-energy X-ray absorptiometry [(DXA), Lunar Idxa, GE Health Care] and stature was measured using a stadiometer (Holtain Ltd., Crymych, Wales). Participants were each familiarised by the same researcher, using standardised language, to the Borg 6–20 (‘no exertion at all’–‘maximum exertion’) Rating of Perceived Exertion (RPE) scale [20] and completed walking on a motorised treadmill (Pulsar, h/p/cosmos, Nussdorf-Traunstein, Germany) at a 1% gradient, to determine a self-perceived moderate-intensity speed (RPE 12–14, ‘somewhat hard’). This speed was used during the walking breaks in the relevant condition described below. A moderate-intensity walking speed was used in line with the Qatar physical activity guidelines that focus on increasing moderate-intensity physical activity [9]. Finally, participants were familiarised using standardised language from the same researcher with the cognitive performance assessment tasks and visual analogue scale for fatigue (VAS-F) [21].

### Experimental conditions (visits 2 and 3)

Participants attended in the morning (~08:00) following an overnight fast and minimised activity while travelling to the laboratory (e.g. travelled by car). The two conditions were:

- Uninterrupted sitting (SIT): participants remained seated throughout the experimental period.
- Sitting with walking breaks (WALK): sitting was interrupted with moderate-intensity walking [RPE 12–14; treadmill belt speed (minimum–maximum) was 6.0 (5.0–8.3) km.h^-1^] for 3-min every 30-min, accumulating nine bouts (27-min) in total (see Fig 1).

A high glycaemic index standardised mixed breakfast meal (see Table 2) at 0-h and a moderate glycaemic index snack (see Table 2) at 3-h was consumed providing 30% and 20%, respectively, of estimated daily energy needs for each participant, which was calculated using validated equations with a physical activity factor of 1.4 applied to represent a sedentary day [22]. The glycaemic index of the meals was calculated using methods described previously [23]. Participants were permitted to void when needed. The toilets were located in the laboratory, and participants were transported in a wheelchair to the toilets on each occasion to limit movement. Water was provided ad libitum during the first condition and the volume consumed was replicated during the second condition. Participants were supervised throughout the conditions to ensure adherence to the protocol, and were permitted to watch DVDs, read, talk, or work on a laptop when seated. During sitting periods, participants were asked to minimise excessive movement and remained seated.

**Table 2:** Standardised breakfast and snack composition. Data is presented as mean ± standard deviation.

Content	Breakfast	Snack
	Cornflakes with whole milk & a croissant	Lusine Chocolate puff
Carbohydrate (g)	80 ± 11	35 ± 5
Fat (g)	19 ± 3	14 ± 2
Protein (g)	17 ± 2	6 ± 1
Energy (kcal)	570 ± 78	285 ± 39
Carbohydrate (%)	57	48
Fat (%)	28	44
Protein (%)	15	8
Glycaemic index	70	67

### Cognitive testing

During each experimental condition, cognitive function tasks were performed at baseline (-15-min), 2.5-h and 5-h as per Fig 1. Cognitive function was assessed using the Computerised Mental Performance Assessment System (COMPASS; Northumbria University, Newcastle Upon Tyne, UK). This testing system delivers a bespoke collection of tasks, with fully randomised parallel versions of each task delivered at each assessment for each individual. Tasks were presented on a laptop PC with responses made either via mouse and cursor, or the keyboard number pad and space bar. The selection of standard, classic, cognitive tasks were specifically chosen to provide a broad assessment across all cognitive domains, including episodic memory, working memory, attention and executive function [24]. This approach has been employed successfully in previous cognitive-related research [25–27]. The tasks are described within Table 3, in order of completion, with this order held constant within and between participants and conditions.

**Table 3:** Computerised Mental Performance Assessment System (COMPASS) task descriptions.

Task (duration)	Cognitive domain	Description
Serial-3 subtractions (2-min)	Executive function & working memory	A starting number between 800 and 999 appeared on the screen and participants were instructed to count backwards as quickly and as accurately as possible from this number in threes, using the linear number keys to make their response. Responses were cleared when the ‘enter’ key was pressed. Participants were only shown one number on screen and the rest of the numbers were generated by subtracting from the previous number in their head. In the case of incorrect responses, subsequent responses were scored positively if they were correct in relation to the new number. This timed task was scored for total responses, correct responses, and number of errors.
Serial-7 subtractions (2-min)	Executive function & working memory	This task is identical to the serial 3s subtraction task except that it involves the serial subtraction of 7s.
Simple reaction time (50 stimuli)	Attention & vigilance	An upwards-pointing arrow was displayed on the screen at irregular intervals. Participants responded by pressing the response button as quickly as they could as soon as they saw the arrow appear. The inter-stimulus interval varied randomly between 1 and 3.5 s. Reaction time (ms) was recorded.
Rapid visual information processing (5-min)	Attention & vigilance	An arrow appeared on the screen pointing to the left or to the right. Participants responded with a left or right key press corresponding to the direction of the arrow. There was a randomly varying inter-stimulus interval of between 1 and 3 s for a total of fifty stimuli. Accuracy (% overall) and mean reaction time (ms) overall, and for correct responses were recorded.
Choice reaction time (50 stimuli)	Attention & vigilance	Participants monitored a continuous series of single digits (1–9) for targets of three consecutive odd or three consecutive even digits. The digits were presented on the computer screen at the rate of 100 per minute in a pseudo-random order, with the participant responding to the detection of a target string with a space bar key press. Eight target strings were presented in each minute. The task was scored for accuracy (% overall), average reaction time (ms) for correct responses, and number of false alarms.
Stroop (60 stimuli)	Attention, vigilance & executive function	Words describing one of four colours (“RED”, “YELLOW”, “GREEN”, “BLUE”) were presented in different coloured fonts in the centre of the computer screen. The participant pressed one of four coloured response buttons in order to identify the font colour (e.g., if the word ‘GREEN’ was presented in a blue font, the correct response would be to respond with the blue button). The presented words were either “congruent” (word and font are the same colour) or “incongruent” (word and font are different colours) and were presented in a random order. This task was scored for accuracy (% overall, % congruent, % incongruent) reaction time (ms) overall, overall correct responses, overall congruent responses, overall incongruent responses, congruent correct responses, incongruent correct responses and interference reaction time (difference in reaction time for congruent and incongruent stimuli). Responses below 150 ms do not register.

### Visual analogue scale for fatigue (VAS-F)

The VAS-F consists of eighteen items relating to the subjective experience of fatigue. Participants were required to circle a number relating to how they currently feel along a visual analogue line that extends between two extremes (e.g. ‘not tired at all’ to ‘extremely tired’). Each line is 100 mm in length and therefore the scores fall between 0 and 100. There are two subscales; fatigue (items 1–5, and 11–18) and energy (items 6–10). Scoring was performed by summing the scores for each item and dividing by eighteen. The scores for the energy items (6–10) were reversed. This scale has been validated with adults aged 18–55 years [21].

### Statistical analyses

Statistical analyses were performed using the Statistical Package for the Social Sciences (SPSS) version 24 (IBM, SPSS Inc, Chicago, IL, USA). Initially, descriptive statistics were generated, and normality checked using quantile-quantile (Q-Q) plots [28]. Descriptive statistics are reported as mean ± standard deviation (SD) and range (minimum–maximum). Linear mixed models (LMM) were used to determine if there were any main effects of condition (SIT and WALK) and time (-15-min, 2.5-h and 5-h), in addition to a condition x time interaction effect for the COMPASS and VAS-F outcomes. Fixed (condition and time) and random (participants) effects for the LMM were fit for each dependent variable [29]. Baseline values for the COMPASS and VAS-F were entered as covariates. The most appropriate model was chosen using the smallest Hurvich and Tsai criterion (AICC) [30] in accordance with the principle of parsimony. The least squares mean test provided pairwise comparisons between the fixed effects. Step down Hommel p value adjustments were used for post hoc analysis in the event of a significant main and/or interaction effect [31]. Normality and homogeneity of variance of the residuals were checked using Q-Q plots, and scatter plots respectively, and deemed plausible in each instance. Significance was accepted as p ≤ 0.05.

## Results

Sixteen participants were recruited for the study with three withdrawing prior to commencing the experimental procedures and two being unable to complete all three visits. There was no missing data for the eleven participants who completed the study.

There were no significant differences in baseline values for any of the COMPASS tasks between SIT and WALK (p ≥ 0.13). The results for the COMPASS and VAS-F in SIT and WALK at baseline (-15-min), 2.5-h and 5-h are shown in Table 4. Significant fixed effects (condition and time) and interaction effects (condition x time) are indicated in Table 4.

**Table 4:** Scores from the COMPASS cognitive tasks and VAS-F at baseline (-15-min), 2.5-h and 5-h in SIT and WALK. Data are presented as median (minimum–maximum).

	SIT			WALK		
	Baseline	2.5-h	5-h	Baseline	2.5-h	5-h
Serial-3 total responses	12 (6–36)	12 (7–41)	14 (6–46)	15 (1–28)	13 (4–37)	15 (6–35)
Serial-3 errors	2 (0–23)	3 (0–26)	2 (0–23)	0 (0–32)	1 (0–21)	2 (0–27)
Serial-7 total responses	11 (2–18)	8 (2–30)	10 (2–18)	7 (4–14)	9 (3–19)	8 (3–29)
Serial-7 errors	4 (0–18)	2 (0–30)	2 (0–18)	1 (0–14)	1 (0–19)	2 (0–28)
SRT (ms)	419.2 (361.5–1371.3)	500.3 (337.9–765.1)	486.0 (403.9–1351.5)	421.6 (313.3–790.0)	416.4 (319.1–670.5)	436.5 (366.3–699.9)
CRT correct	98 (90–100)	98 (90–100)	96 (90–100)	98 (92–100)	98 (94–100)	98 (90–100)
CRT (ms)	530.9 (495.4–861.9)	550.7 (464.8–920.7)	574.3 (446.7–965.0)	511.7 (430.2–720.6)	564.5 (430.6 (810.9)	524.2 (409.9–1359.7)* ^†^
RVIP correct	10 (0–30)	13 (3–30)	8 (0–25)	23 (3–45)	23 (5–33)	20 (5–33)
RVIP RT (ms)	590.1 (432.7–669.0)	527.9 (344.2–696.0)	541.2 (364.2–603.6)	486.8 (361.0–615.2)	521.5 (440.7–599.0)	523.9 (408.8–783.0)
RVIP false	22 (0–193)	45 (0–99)	42 (3–130)	11 (0–90)	14 (0–107)	20 (0–91)
Stroop correct	100 (58–100)	98 (50–100)	98 (57–100)	100 (52–100)	100 (52–100)	100 (13–100)
Stroop RT (ms)	1091.6 (924.9–1965.1)	1212.2 (988.8–2725.9)	1000.2 (154.3–1856.3)	1112.8 (871.8–1647.1)	985.3 (805.6–1745.1)	1010.9 (821.7–1845.4)
Stroop RT (ms) congruent	1129.0 (859.8–1558.2)	1084.7 (754.7–1516.9)	1019.0 (824.9–1569.9)	1013.2 (915.9–2090.6)	1009.6 (925.8–3431.3)	1010.0 (824.1–1678.7)
Stroop RT (ms) incongruent	1096.7 (984.6–2419.0)	1147.6 (870.2–2177.0)	1025.0 (864.7–3542.8)	997.3 (893.6–1586.2)	999.8 (858.5–1376.5) ^#^	1018.1 (820.2–2419.0)
Stroop RT (ms) interference	-74.8 (-902–37)	-122.8 (-727.1–118.7)	-41.5 (-1972.9–17.0)	19.4 (-213.6–718.2)	45.3 (-28.5–2059.7)	21.4 (-740.3–231.9)
VAS-F	34 (0–93)	39 (10–86)	44 (20–89)	25 (0–54)	39 (0–58)	52 (0–78)

* Significant fixed effect for condition

† significant fixed effect for time

# significant interaction effect (condition x time) (p < 0.05); SIT = uninterrupted sitting; WALK = breaking up sitting with walking

### Condition effects

There was a significant condition effect for CRT (f = 26.7, p = 0.007). On average CRT was 101 s (95% CI = -47 to -156 s) quicker in WALK compared to SIT.

### Time effects

There was a significant time effect for CRT (f = 15.5, p = 0.01). On average CRT was 134 s slower at 5-h compared to baseline (p = 0.006; 95% CI = -64 to -203 s), and 114 s slower at 5-h compared to 2.5-h (p = 0.01; 95% CI = -44 to -183 s).

### Condition x time effects

There was a significant interaction effect for RT in the Stroop incongruent task (f = 10.0, p = 0.03). On average RT was 210 s quicker at 2.5-h in WALK compared to SIT (p = 0.01; 95% CI = -76 to -346 s).

## Discussion

The main findings in the present study were that breaking up prolonged sitting with short (3-min) frequent (every 30-min) moderate-intensity walking improves attention and executive function in sedentary Qatari females. Other cognitive tasks and fatigue remained unchanged. Therefore, rejecting the hypotheses.

Reaction time (CRT and Stroop) was improved during WALK in the present study. Conversely, light-intensity walking every 30-min in older (45–75 y) adults [32], and young adult males under sleep restriction [33] did not improve cognitive function. The contrasting cognitive function changes in the present data are likely due to a quicker walking speed (range: 5.0–8.3 km.h-1), unlike the 3.2 km.h^-1^ employed elsewhere (33, 34), and the fact that participants were not sleep deprived, as in previous work [33]. Higher intensity physical activity (i.e. a quicker walking speed) may be associated with greater physiological effects (e.g. increased heart rate, catecholamine, brain-derived neurotrophic factor, epinephrine and nor-epinephrine release), which may improve cognitive function to a greater extent than lower-intensity physical activity, particularly when there is a delay between the walking break and cognitive task [34]. Nevertheless, a single 6-min high-intensity interval training bout (including exercises such as squatting, skipping, jumping) to break up sitting time over 3-h in students found no improvement in the Stroop colour test [35]. Short-term increases in memory and attention resulting from treadmill walking may have a delayed effect [34], and could explain why performing cognitive tasks during treadmill walking does not improve cognition [36]. Additionally, the shorter intervention period (i.e. 3-h compared to 5-h in the present study), less frequent physical activity bouts and lower volume of physical activity [i.e. a single 6-min bout compared to 3-min every 30 min (27-min total) in the present study] could explain differences in these findings. Based on these findings, it appears that frequent physical activity breaks of a moderate-intensity may be required to improve cognitive function. Furthermore, this data suggests that a break is required following treadmill walking prior to cognitive function being assessed and that cognitive tasks may be impaired acutely whilst walking on a treadmill. However, future research should evaluate the cognitive performance responses during treadmill walking after a period of familiarisation with such a workstation e.g. after several weeks [12]. It may be more cost effective for an organisation to provide a limited number of ‘shared’ treadmills within the workplace as opposed to providing every employee with an active-workstation. Within Qatar, females may feel more comfortable if they could break up their sitting by walking on a treadmill in a private ‘female-only’ room for cultural reasons. At the simplest level, if personal preference allowed, Qatari females could simply walk around their air-conditioned offices at an appropriate RPE based walking speed. However, further work examining the long-term effects of breaking up prolonged sitting on multiple cognitive domains are necessary, before recommendations at a population level can be made.

Breakfast consumption had no effect on cognitive performance in the present study (i.e. there was no difference between baseline and 2.5-h). Breakfast consumption has been shown to both improve [37, 38] and worsen [39] cognitive function. Subsequently, future studies should consider the effects of the inclusion and composition of breakfast on cognitive performance [39] and whether this could affect cognitive responses to breaking up prolonged sitting.

Although not directly quantified within the present study, it is important to consider what mechanisms could be associated with the intervention mediated enhanced cognitive function. Prolonged sitting mediated suppression of the sympathetic nervous system activity has been shown, with associated increases in fatigue and plasma dihydroxyphenylglycol (DHPG) alongside concurrent decreases in heart rate and plasma dihydroxyphenylalanine (DOPA) [40]. Given prolonged sitting decreases [7] and breaking up prolonged-sitting [(using a similar intervention to that utilised in the present design [7]] can increase cerebral blood flow, it is proposed that proliferation of cerebral blood flow may enhance/stimulate central nervous system functioning, principally through delivering more glucose to the brain [41]. Future deductive research should focus on exploring the neurobiological mechanisms associated with cognitive function during prolonged sitting and the subsequent favourable responses seen with the interruption of such sedentary behaviours.

There was no effect of breaking up sitting time on fatigue in the present study. Contrastingly, 5-min of moderate-intensity walking every 60-min for 6-h improved mood and decreased levels of fatigue in sedentary adults [40]. Similarly, in older (45–75 y) adults [32], and young adult males under sleep restriction [33] 3-min of light-intensity walking every 30-min decreased fatigue and enhanced alertness, respectively. Furthermore, a recent study employing a 6-min high-intensity interval training test (e.g. squatting, skipping, jumping) to break up sitting time over 3-h in students found significant improvements in vigour (measured using the short version of the Profile of Mood States) [35]. The equivocal nature of this data [15, 32, 33, 35] compared to the present study could be due to differences in fatigue measurement tools employed, participants being sleep restricted [33], and variation in the frequency, intensity and duration of the physical activity performed. Participants in the present study were sedentary females with high levels of self-reported sedentary behaviour. The moderate-intensity walking breaks could have caused some subjective fatigue if they were not used to walking at such speeds, even though a familiarisation session was performed. Medium and long-term interventions should be conducted to examine the effects of moderate-intensity walking breaks on fatigue, which could then inform public health and workplace recommendations.

The main strength of this study is the evaluation of a female Qatari sample who present unique environmental and cultural barriers to reducing sitting time. However, the study is limited by its acute nature, meaning that the chronic responses to breaking up prolonged sitting cannot be inferred. Furthermore, as stated previously, the mechanisms that could explain cognitive function responses to such an intervention were not studied and should be explored in future research. Lastly, a moderate-intensity walking pace was used in the present study. It could be argued that light-intensity walking could have greater ecologically validity. The effects of light-intensity walking breaks on cognitive function in Qatari females should thus also be investigated.

## Conclusion

This study suggests that interrupting prolonged sitting with moderate-intensity walking acutely improves certain aspects of cognitive function without affecting fatigue in Qatari females. In a population that falls considerably short of the recommended physical activity guidelines and has a high amount of sedentary behaviour, this ecologically valid intervention, which conforms to Islamic cultural beliefs and can easily be performed at work while wearing Islamic traditional clothing (i.e. Abaya). Long-term studies, employing multiple cognitive domains testing and neurobiological measurements, are needed to determine the chronic cognitive function effects of walking breaks before general recommendations at a population level can be made.
